# Co-creation of an ICT-supported cancer rehabilitation application for resected lung cancer survivors: design and evaluation

**DOI:** 10.1186/s12913-016-1385-7

**Published:** 2016-04-27

**Authors:** Josien G. Timmerman, Thijs M. Tönis, Marit G. H. Dekker-van Weering, Martijn M. Stuiver, Michel W. J. M. Wouters, Wim H. van Harten, Hermie J. Hermens, Miriam M. R. Vollenbroek-Hutten

**Affiliations:** Roessingh Research and Development, Telemedicine group, Roessinghsbleekweg 33b, Enschede, 7522 AH The Netherlands; University of Twente, Faculty of Electrical Engineering, Mathematics and Computer Science, Telemedicine group, Enschede, The Netherlands; The Netherlands Cancer Institute, Amsterdam, The Netherlands

**Keywords:** Lung cancer, Telehealthcare, Rehabilitation, User-centered design approach, Cancer survivorship

## Abstract

**Background:**

Lung cancer (LC) patients experience high symptom burden and significant decline of physical fitness and quality of life following lung resection. Good quality of survivorship care post-surgery is essential to optimize recovery and prevent unscheduled healthcare use. The use of Information and Communication Technology (ICT) can improve post-surgery care, as it enables frequent monitoring of health status in daily life, provides timely and personalized feedback to patients and professionals, and improves accessibility to rehabilitation programs. Despite its promises, implementation of telehealthcare applications is challenging, often hampered by non-acceptance of the developed service by its end-users. A promising approach is to involve the end-users early and continuously during the developmental process through a so-called user-centred design approach. The aim of this article is to report on this process of co-creation and evaluation of a multimodal ICT-supported cancer rehabilitation program with and for lung cancer patients treated with lung resection and their healthcare professionals (HCPs).

**Methods:**

A user-centered design approach was used. Through semi-structured interviews (*n* = 10 LC patients and 6 HCPs), focus groups (*n* = 5 HCPs), and scenarios (*n* = 5 HCPs), user needs and requirements were elicited. Semi-structured interviews and the System Usability Scale (SUS) were used to evaluate usability of the telehealthcare application with 7 LC patients and 10 HCPs.

**Results:**

The developed application consists of: 1) self-monitoring of symptoms and physical activity using on-body sensors and a smartphone, and 2) a web based physical exercise program. 71 % of LC patients and 78 % of HCPs were willing to use the application as part of lung cancer treatment. Accessibility of data via electronic patient records was essential for HCPs. LC patients regarded a positive attitude of the HCP towards the application essential. Overall, the usability (SUS median score = 70, range 35–95) was rated acceptable.

**Conclusions:**

A telehealthcare application that facilitates symptom monitoring and physical fitness training is considered a useful tool to further improve recovery following surgery of resected lung cancer (LC) patients. Involvement of end users in the design process appears to be necessary to optimize chances of adoption, compliance and implementation of telemedicine.

## Background

Lung cancer is the most commonly diagnosed malignancy among adults worldwide, as well as the leading cause of cancer-related death [1]. Approximately 80 % of lung cancer patients will be diagnosed with non-small cell lung cancer (NSCLC), and around 25 % will present with early-stage, operable disease [2]. Curative lung resection is the preferred treatment for early-stage NSCLC [3], but is associated with a considerable symptom burden such as pain and fatigue, as well as decay of lung function, cardiorespiratory fitness, and Quality of Life (QoL) [4–8]. After hospital discharge, monitoring of self-reported symptoms and disability encountered in daily life is limited to a few, planned consultations. It is reported that clinicians systematically underestimate patients’ symptoms, increasing the risk that crucial symptoms are overlooked [9]. In addition, NSCLC patients report high levels of supportive care needs [10], and often feel insecure about their health status and do not know what to do to improve their recovery [11]. These issues call for the development of new methods that enable better (objective) monitoring of the patients, as well as tools that increase the level of self-management of the patient in order to optimize post-surgery recovery of health status in NSCLC patients.

A promising method to improve survivorship care is the use of telehealthcare. Telehealthcare is ‘*the provision of personalized healthcare by a healthcare professional over a distance using Information and Communication Technology (ICT)’* [12]. It is considered instrumental in maintaining good-quality patient care in the shift from inpatient to ambulatory care [13]. Next to that, telehealthcare has several advantages supplemental to face-to-face treatment. For example, it facilitates frequent monitoring of health status and patient-reported outcome measures, and can provide both the patient and healthcare professional with timely and personalized feedback [14–16]. It also supports patients to incorporate behavioural changes directly into daily life, improving their health-related self-management skills. The potential of telehealthcare applications to improve cancer care throughout the entire continuum – including supportive care – has been recognised [17, 18] and various studies showed that telehealthcare applications are acceptable for patients and considered clinically safe [19].

However, the number of tailored applications for resected NCSLC patients is limited, with as far as we know, only two single-component applications reported: a symptom management application [20], and a 6-week light-intensity, home-based exercise program using the Wii Plus [21, 22]. Each of these focusses on a single aspect of support, namely either symptom control or physical fitness. Considering the high number and complexity of supportive care needs, it could be expected that a multimodal intervention would better meet the needs of NSCLC patients.

The primary objective of this study was 1) to develop a multimodal telehealthcare application that aimed at improving post-surgery rehabilitation and physical activity, in close cooperation with resected NSCLC survivors and their healthcare professionals (HCPs), and 2) to evaluate its usability. Through close involvement of the target users we aimed to explore potential new areas of care for lung cancer patients -- from both a HCP and patient perspective -- that might benefit from ICT-supported care. Next to that, we expected that this close cooperation would uncover requirements crucial for adoption early on in the project as to promote adoption and implementation on the long term.

## Methods

**Fig. 1:**
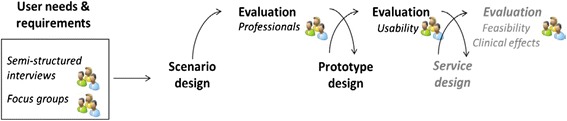
Design approach for the co-creation of our telehealthcare application. The people-figures at the various steps indicate that target users were involved. The steps written in grey indicate future steps in our study

A promising approach, proven to successfully fit ICT-supported services to users’ requirements, is to involve the end-users early and frequently during the developmental process through a user-centred design (UCD) approach [23]. This is an iterative, cyclical process during which design and evaluation phases are alternated. In this way, systems and services can be developed step by step, so that changes in technology and work process can evolve together, and unforeseen challenges can be easily anticipated in future development steps. The followed process is visualized in Fig. 1.

### User needs and requirements

The first aim was to gain insight into the needs and requirements of HCPs and NSCLC patients after resection, regarding the content, the expected benefit on clinical outcome, and contextual aspects of use of a telehealthcare application designed to improve rehabilitation and stimulate physical exercise.

*Semi-structured interviews* were held with HCPs and NSCLC patients based on the framework of the Unified Theory of Acceptance and Use of Technology (UTAUT), since UTAUT has been proven to capture the users’ intentions to accept ICT [24]. First, HCPs from the professional network of the authors MW and MV were approached by email for participation. In turn, participating HCPs were asked for other potential participants, both HCPs and NSCLC patients. Resected NSCLC patients were recruited via participating HCPs and via advertisement on the website of the Dutch patient association for lung cancer. The interviews were performed by the first author (PhD student, human movement scientist and occupational therapist), and lasted between 60 and 90 min. Prior to the interviews the researcher did not have any relationship with the participants, except for one HCP (i.e., the fifth author MW). Interviews took place at the patients’ home or at the workplace of the HCPs. To familiarize participants with telehealthcare and its wide range of possibilities [18, 25], mock-ups were shown with potential functionalities: (1) tailored information about disease and treatment, (2) tailored lifestyle information, (3) ambulant monitoring of health status, (4) web based tailored exercise program, (5) contact with professional by means of ICT (e.g., e-consultation), and (6) contact with fellow lung cancer patients. The content of the mock-ups were based on existing applications that are already available for other chronic diseased populations such as COPD [26, 27]. During the interview needs and requirements regarding content (UTAUT component ‘performance expectancy’), usability/ease of use (‘effort expectancy’), influence of important others on use (‘social influence’), facilitators and barriers for use (‘facilitating conditions’), and intention to use the service were assessed.

Subsequently *focus groups* were held with HCPs in which the functionalities derived from the interviews were discussed, and specific requirements for both the technology and implementation in every day practice were defined in more detail. All HCPs approached for the interviews were contacted again for participation in the focus groups. Additionally, HCPs from the Netherlands Cancer Institute (NKI), a designated cancer center in the Netherlands, known to be involved in the post-surgery care for lung cancer patients were asked for participation by the first author. The focus groups were performed by the authors JT, TT (PhD student, background in biomedical engineering) and MD (PhD, human movement scientist). The focus groups were audio recorded and transcribed verbatim with participants’ permission. Data were arranged in themes using the UTAUT components by authors JT and MD.

As a third step, a *scenario* was described, validating the requirements drafted from the interviews and focus groups. Scenarios are stories describing the activities, in detail, of persons when using the envisioned ICT application with a specific goal and within a specific context [28, 29]. The scenario described: 1) a NSCLC patient using the telehealthcare application as part of her lung cancer treatment and recovery process, and 2) a visualization of the measurement protocol for home-based symptom monitoring. The written scenario was sent by email to ten HCPs employed at the NKI who are involved in the post-surgery care for lung cancer patients. Professionals were instructed to read the scenario in detail and to write comments, both positive and negative, as tracked changes in the text of the scenario. Comments were send back to the first author via email.

### Prototype design

Authors JT and TT selected relevant requirements from the interviews, focus groups and scenario comments. Authors MV and MD validated the selection. Considerations for requirement selection included (in order of importance): 1) context of use of the application, including aim for which the application will be used, overlap or integration with existing services and people involved, 2) technical feasibility and time available to realize the requirement, 3) the number of participants who mentioned it, and 4) factors influencing future dissemination possibilities in multiple institutions and other cancer diagnoses. As an example for our study, the NKI already hosted an interactive electronic patient record (EPR) including tailored information [30]. This contextual information was taken into account during development to optimize adoption and implementation of the application in the NKI. From the selected requirements, technical requirements were derived after which the first prototype was developed.

### Evaluation of usability

#### Participants

HCPs from various disciplines and healthcare institutions were recruited, using the professional network of the authors MS, MW, and MV. Colleagues of participating HCPs were also asked for participation. NSCLC patients were recruited via HCPs who participated in this evaluation. Included NSCLC patients were 18 years or older, treated with lung resection within the previous two years (with or without adjuvant treatment), and had no recurrence of cancer at enrolment. Only HPCs who treated resected NSCLC patients at the moment of enrolment, were included.

#### Procedures

The interviews were performed by the first author at the patients’ home and at the HCPs workplace. Each session lasted between 30 and 60 min. First, the aim and procedure of the session were explained after which the participants signed informed consent. Next, participants received a short introduction of the aim and content of the telehealthcare application, together with a user manual of the modules. Then they interacted with the modules by completing several predetermined tasks while verbalizing their thoughts out loud (‘thinking-aloud’ method) [31]. After completion, semi-structured interviews were performed to evaluate the content (UTAUT component ‘performance expectancy’), interface (visual design), ease of use (‘effort expectancy’), and intention to use the modules. All interviews were audio-recorded and transcribed verbatim with participants’ permission. Lastly, the participant completed the System Usability Scale (SUS) questionnaire. The SUS consists of ten statements to which the participant can agree or disagree on a 5-point scale [32]. All the responses are summed and multiplied with 2.5, resulting in a score between 0 and 100, with a higher score meaning better usability. Cut-off scores of ≤ 50, 51–69, and ≥ 70 were considered ‘not acceptable’, ‘OK’ (i.e., ‘moderately acceptable’) and ‘acceptable’ usability, respectively as suggested by Bangor et al. [32].

#### Data-analysis

Comments on usability issues were extracted from the transcripts and classified by authors JT and MD through an inductive approach using the following classifications [33]:

- content & information: missing content such as relevant outcome variables, exercises, functionalities or written information.
- navigation & structure: problems with navigating through the portals. For example, location where information is located on the portal.
- design & presentation: representation of the available data and information. For example, color use, graphs, amount of text on a page or smartphone.
- ambulant devices: remarks regarding the use of the sensors (that is smartphone, activity monitor, heart rate sensor and oxygen saturation sensor).

In this early phase of evaluation the focus is on the reported points of improvements only, with the aim to generate redesign input [34]. Therefore, only the points of improvement are reported in this article. These comments were further classified as being critical, serious, or minor using the following definitions [34]:

- A *critical* problem prevented participants from completing tasks or was reported by all participants.
- A *serious* problem severely increased task completion time or was reported by ≥ 50 % of the participants. However, a serious problem did not prevent a participant from completing the task eventually.
- A *minor* problem increased task completion time slightly or was reported by < 50 % of participants.

## Results

### User needs and requirements

#### Semi-structured interviews

Ten NSCLC patients (mean age ± sd = 62 ± 11 years, 70 % female, mean ± sd time since resection = 6 ± 3 months) treated with lung resection and six HCPs involved in post-surgery care of NSCLC patients (2 pulmonary rehabilitation specialists, 1 pulmonologist, 1 thoracic surgeon, 1 physiotherapist, 1 nurse practitioner; 50 % female) from four different hospitals participated in the interviews.

All participants expected that a telehealthcare application would be of clinical benefit for resected NSCLC cancer patients. In particular, HCPs valued the application as a method to improve quality of current care, while patients considered it as a way to decrease their insecurity about experienced symptoms, their recovery, and healthy behaviour.

*“That fear [for recurrence], if you can put an end to that by surveillance, I would do anything. Even if it is not OK, that you know this in time…get a hint”* (Patient 2, female, 59 years).

*“I do not need to measure it [lung capacity] every day, but once a month, something like that. Especially when regular visits to the lung physician have ended, then I would like to know. To be reassured”* (Patient 7, male, 48 years).

Other expected benefits were improved patient-HCP communication -- since HCPs will have better insight in relevant issues -- and improved accessibility to care provided by a specialised cancer centre while being at home.

**Fig. 2:**
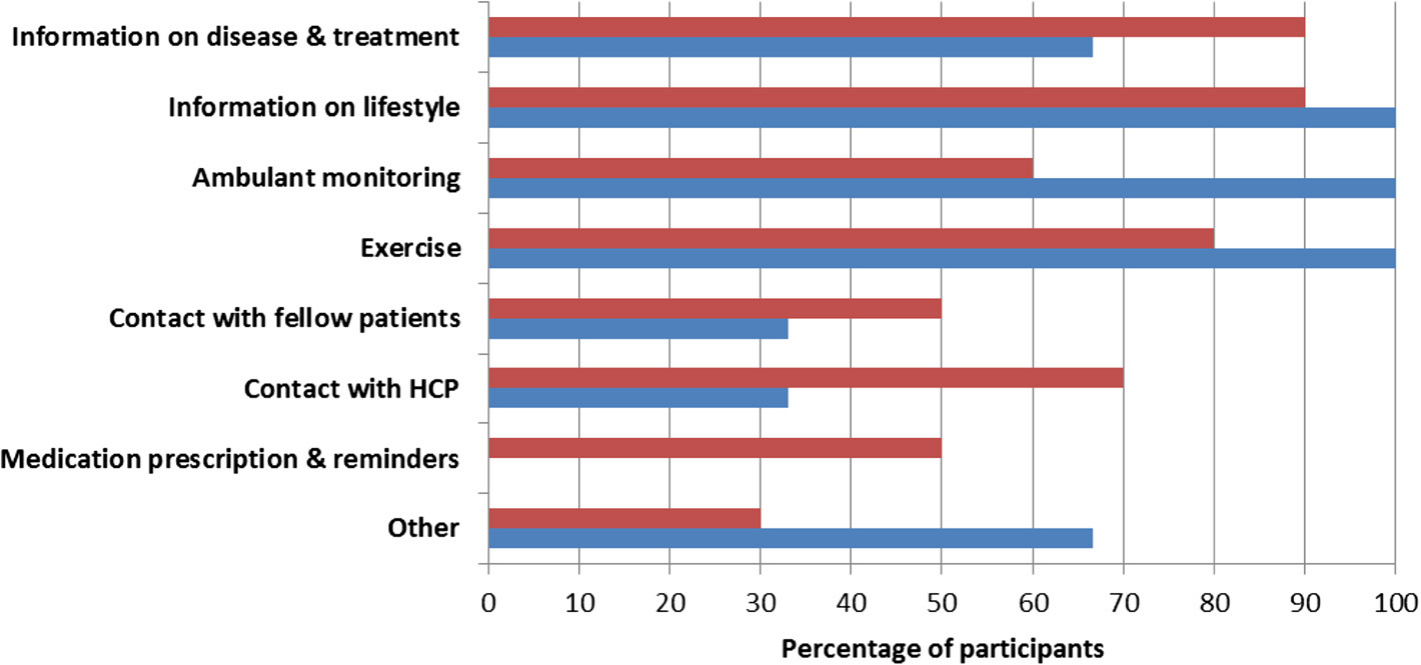
Functionalities for the telehealthcare application reported by resected LC patients and HCPs. Red bars = resected LC patients (*n* = 10); blue bars = healthcare professionals (*n* = 6). Abbreviations: HCP = healthcare professional

Figure 2 summarizes the functionalities that were regarded useful by the participants to improve survivorship care following lung resection. The majority of both patients and professionals considered ambulant monitoring of health status, tailored information about disease, treatment and lifestyle, and a web based tailored exercise program of added value. Additional (“other”) functionalities mentioned were psychological education (*n* = 4), support for family or other caregivers (*n* = 1), and treatment of pain (*n* = 1). Although HCPs agreed that fellow patient contact might be beneficial, they voiced concerns regarding hosting such a service, since this would mean continuous moderation by a HCP to check statements posted by patients. The physicians also reported a negative attitude towards ICT-supported contact between HCPs and patients. They expected that the costs in time and money outweigh the added value if patients are able to contact them by email, chat or web based consultation. In contrast, the physiotherapist and nurse practitioner considered e-consultation as an opportunity to improve quick and easy access to the professional when needed.

For patients, a positive attitude of the HCPs towards the service, as well as feedback from the HCP on results were considered essential to motivate use and compliance*.*

*“That [the attitude of the HCP] will be important. If the physician isn’t motivated, they will not use it, and they will not motivate us, if they aren’t enthusiastic.”* (Patient 3, female, 58 years).

Use of smartphones and computers were seen as barriers. However, this could be solved by good usability of the service and adequate instruction. All patients reported willingness to wear monitoring devices and complete questionnaires, as long as this would not restrict daily functioning and would be of clinical value for their HCP. For HCPs the most important barrier mentioned was the limited time available for preparation and patient consultation.

*“We only have 10 minutes per patient. I am already lucky when I have time to consult the medical record of the next patient. If I have to log on in advance as well… I can’t imagine doing that. That would be too time-consuming”* (pulmonologist, female).

*“You have to connect with the electronic patient record, so you can consult all information at once. If you have to switch between systems during a patient consult, that doesn’t work very well”* (thoracic surgeon, male).

Therefore, integration of the service with existing electronic patient records, as well as adequate summary of the measured outcome parameters into a coherent overview were regarded critical and were further defined during the focus groups.

#### Focus groups

Five HCPs (thoracic surgeon, pulmonologist, two physiotherapists, nurse, 60 % female, all employed at the NKI,) participated in the focus groups. The HCPs emphasized the importance of a flexible, modular system, that allows for quick and easy adjustment to various patients and patient groups. HCPs selected three main treatment modules: ambulant symptom monitoring, a web based exercise program, and tailored information on disease and treatment. Since tailored information on disease, treatment and an active lifestyle were already available as part of the EPR in the NKI [30], this module was not developed further as part of our telehealthcare application. The content of the other two selected modules was discussed and defined as follows:

- *Ambulant symptom monitoring* aims to provide insight in the rate of recovery after surgery, by monitoring self-reported symptoms and physiological parameters at home and over time. For this, monitoring should integrate self-reported symptoms (pain, dyspnea and fatigue) with physiological parameters (heart rate and oxygen saturation).
- The *web based exercise module* should enable the patients to recondition at home using a personalized set of exercises, which will be remotely supervised. Physiotherapists need to be able to easily select a specific set of exercises suited for resected lung cancer patients, and adapt training level and program from a distance.

#### Scenario evaluation

Five HCPs provided feedback on the written scenario; a rehabilitation physician, a thoracic surgeon, two physiotherapists, and a nurse. All were optimistic about the telehealthcare application as described in the scenario, and expected that it would benefit recovery following lung resection. The feedback given by the professionals primarily concerned implementation issues. For example, one of the professionals questioned the feasibility of ambulant monitoring in this population: *“I find this a good idea. However, I believe this [use of the application] comprises too much ‘activities’, especially in such a pre-post surgery trajectory. I am curious about its feasibility”* (rehabilitation specialist, male). No specific comments were given on the technology described in the scenario.

#### Application requirements

A summary of the selected requirements is given in Table 1. These application requirements were used for the development of the first working prototype of the telehealthcare application.

**Table 1:** Requirements for the telehealthcare service reported by LC patients and HCPs

General requirements	
	General service requirements
	• Integration with existing (hospital) electronic patient records • Flexible service to facilitate individual tailoring • User-friendly for (elderly) patients and HCPs • Helpdesk for ICT-related problems
Ambulant monitoring	
	Monitoring of recovery, perceived symptoms and physical activity
	• High mobility to facilitate independent and home-based use by an elderly population without restricting daily activities • Connect and disconnect sensors on patients’ demands • Parameters: physiological, physical activity, weight, symptoms, pain medication use, experienced QoL and daily disability.
Web based exercise	
	Promote physical activity and improve physical fitness pre- and post-surgery
	• Quick and easy selection of exercises and weekly program • Minimally once face-to-face contact with healthcare professional • Supervised and supported by healthcare professionals (from a distance) • Individually tailored based on patient-reported difficulty of performance of exercises
Data access and representation	
	Facilitate adequate data access and interpretation
	• Integration of outcome parameters to facilitate interpretation • Summary of most relevant outcome parameters at top of page • Data available to HCPs prior to planned consultations • Pre-surgery measures as baseline to compare post-surgery recovery

The requirements reported in this table are summarized from the interviews, focus groups and scenario evaluation

### Prototype design

The telehealthcare application is a modular system, consisting of two treatment modules. For the first evaluations, the modules will run independent from each other. The modules run from the Continuous Care and Coaching Platform (“C3PO”), hosted at the research institute [35].

**Fig. 3:**
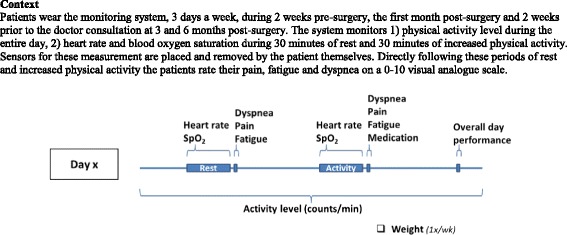
Example monitoring protocol for monitoring health status and recovery in operable lung cancer patients. *Abbreviations:  SpO2 = blood oxygen saturation*. Context: Patients wear the monitoring system, 3 days a week, during 2 weeks pre-surgery, the first month post-surgery and 2 weeks prior to the doctor consultation at 3 and 6 months post-surgery. The system monitors 1) physical activity level during the entire day, 2) heart rate and blood oxygen saturation during 30 minutes of rest and 30 minutes of increased physical activity. Sensors for these measurement are placed and removed by the patient themselves. Directly following these periods of rest and increased physical activity the patients rate their pain, fatigue and dyspnea on a 0-10 visual analogue scale.
- Web based exercise module
  The web based exercise module consists of a patient portal and a professional portal, both accessible through the internet. The starting point of development was an existing web based exercise module, developed and validated as part of the CoCo portal for COPD patients [36]. In close cooperation with physiotherapists of the NKI, the content of the existing webportal was adapted based on selected requirements and exercise protocol deemed relevant for NSCLC patients. The portal is a website hosted at a server at the research centre. The physiotherapist manually selects relevant exercises for the patient from a predefined set. Exercises and exercise intensity are chosen based on the face-to-face intake. The training program is aimed at maintaining or improving overall physical fitness. To minimize the time needed for exercise selection the therapist can define a training level from 0 (easy) to 5 (hard) which results in a pre-selection of exercises corresponding to this training level. Each exercise is illustrated by a movie with spoken instructions, and supported by written text (Fig. 4). The patient can access the training program online via the patient portal, and perform the exercises independently at home. The patient reports the number of exercises performed, the number of exercises successfully completed, and the experienced difficulty of the exercise on a scale from 0 (not difficult at all) to 10 (extremely difficult). After completion of an exercise, patients are asked to indicate whether intensity or difficulty for that exercise could be higher in the training program for the next 4 weeks. This information is summarized in a feedback report available to both the therapist and the patient. Based on this report, the therapist will adjust the training level and select relevant exercises each month. When needed, the patient can select an “emergency” button at the web portal, which results in a standardized email sent to the responsible professional with instructions for the HCP to contact the patient.

- Monitoring system
  The monitoring module is a combined system consisting of an Android smartphone application and an internet webportal accessible via internet. The monitoring system of the patient consists of an android smartphone and three on-body sensors, i.e., accelerometer, heart rate sensor and an oxygen saturation sensor. The sensors transmit the measurement data wirelessly to the smartphone over a Bluetooth connection. The smartphone stores the data and transmits it to the C3PO server - using a secure connection - at set time intervals. The smartphone is used as input device for answering questions about dyspnea, fatigue, pain, medication use and type of activity performed. Once a week the weight of the patient is asked as input. When connection with a sensor is lost or the data are of non-acceptable quality, the smartphone will give instructions on how to improve data quality. To facilitate integration of the symptom scores with physiological parameters, a predefined monitoring protocol is used. Figure 3 describes an example of the monitoring protocol established with HCPs from the NKI. The gathered data are summarized into graphs, combining symptoms with activity intensity. The data are accessible for both patients and HCPs via a web portal. The web portal is integrated with existing EPRs at the hospital using a single sign on, to improve insight in the change of health status and recovery process. On the portal, the graphs most relevant for clinical care are shown first and can be viewed on one page. To increase security, no personal information is stored in the C3PO platform that can directly link the data with a user (e.g., no name, social security number or address information). In the C3PO platform users are identified by a system-id and patient-id. Only within the EPR, accessible within the hospital, the system can link these id's with the real user.

**Fig. 4:**
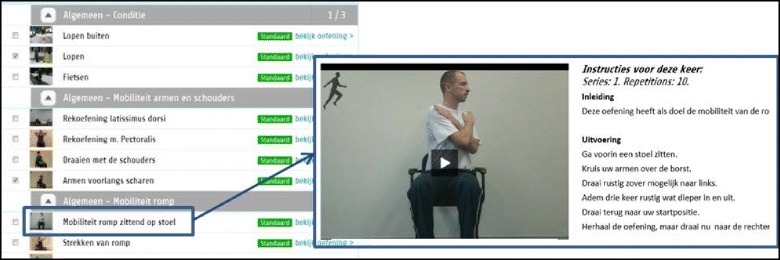
Screenshot of the web based exercise module

### Usability

Seven resected NSCLC survivors (mean ± sd age = 64 ± 9; 57 % female; mean ± sd time since diagnosis = 9 ± 7 months), and 10 HCPs (6 physiotherapists, 2 thoracic surgeons, 1 pulmonologist, 1 oncology nurse; 40 % female) from two different hospitals participated in the usability study.

#### System usability score

Based on the defined cut-off scores for the SUS, moderate to high acceptable usability scores were given for the symptom monitoring module (median: 69; range patients 68–95; range HCPs 63–78). For the web based exercise module usability was more variable, ranging from non-acceptable (*n* = 4) to acceptable (*n* = 5) (median: 70; range patients: 35–80; range HCPs: 45–80).

#### Interviews

All participants voiced positive intentions to use the symptom monitoring module, while only half of participants felt this way about the web based exercise module. In total, 75 usability issues were identified; of which 10 were critical, 25 serious, and 40 were minor issues. HCPs reported 63 % of these issues. The issues critical for efficient use and long term adoption will be reported here.

##### Symptom monitoring

*Design & Presentation* – 21 (4 critical) usability issues were reported. Both HCPs and patients experienced the visualisation of results as “cluttered” with a lot of numbers and dots, making interpretation of the data, especially for patients, somewhat problematic.

*“I don’t think this is very clear with all those dots and numbers”* (patient 2, 61 years, female).

Missing graph legends and introductory texts at each page further hampered quick interpretation of the graphs.

*Content & Information –* Seven (3 critical) usability issues were identified. Both HCPs and patients lacked detailed information on pain medication use (i.e., medication category and amount used on a measurement day). Next to that, HCPs disagreed on the relevancy of the outcome measures included. For example, fatigue was regarded non-relevant by surgeons, but clinically relevant by pulmonologists and the oncology nurse.

*“I don’t think fatigue is useful; as a surgeon I don’t use this”* (thoracis surgeon, male).

Likewise, surgeons and pulmonologists requested overall daily activity level, while pattern of activity throughout the day was of interest for the oncology nurse.

*“I primarily consider the patient; as a human being. For humans the consequences of daily activity influence how you feel; that you feel pain, dyspnea and fatigue due to too much activity. […] So from my perspective this has added value”* (oncology nurse, female).

*Ambulant devices –* Only two usability issues (none critical) were reported by patients.

No usability issues were reported relating to “Navigation & Structure” or “Other”.

##### Web based exercise

*Design & Presentation* – 20 (3 critical) usability issues were reported. HCPs had difficulty interpreting both training compliance and the results of exercise-related questions (e.g., ‘How difficult was this exercise for you?’), due to faulty visualization of these results in their respective tables. For patients, lack of instruction about using features of the portal (for example difficulty playing exercise video) was rated critical. Both patients and HCPs experienced problems with reading and leaving new messages. For example, it was unclear which of the messages was new (HCPs) or where to click to send a new message (patients).

*Content & Information* – 19 issues were detected; 14 by HCPs, 5 by patients. None of these issues were rated critical, that is the issues did not prevent task completion or were reported by a minority of the participants. *Navigation & Structure* – In total four (one critical) issues were identified. For HCPs navigation through the portal in general was rated critical, since all HCPs had difficulty finding one or more components on the site, for example where to select adequate exercises or where to add exercise instructions.

*“So, where can we select the exercises? And the overview? […] This is not logical, I can’t find it”* (Physiotherapist 3, female).

*Other* – Two usability issues (not critical) were reported by the HCPs. Although it did not hamper task completion, HCPs believed that accessibility through smartphone and tablet would promote use and access of the portal in future.

## Discussion

This paper presents the co-creation process of a telehealthcare application for NSCLC patients treated with lung resection. Results of the needs and requirement elicitation study show that both patients and HCPs have positive intentions to use an application that supports post-surgery recovery. For HCPs, improving quality of care was the primary reason to embrace telehealthcare applications. On the other hand, patients valued the increased sense of self-control as a result of insight in patterns of experienced symptoms, as well as easy access to advice and treatment that promotes recovery.

During this first phase of needs and requirement elicitation we presented a broad spectrum of possible applications to participants. Nonetheless, HCPs mainly selected applications that are comparable to existing interventions for lung cancer patients, such as exercise and symptom monitoring [20, 22, 37]. There are several possible reasons why HCPs selected these specific – already available and evidence-based – applications. First, in clinical practice HCPs work with evidence-based treatments to ensure high-quality patient care. This preference for evidence-based practice might have caused the selection of already tested applications [25, 38]. Second, the clinical context of HCPs and their role in the clinical care of lung cancer patients possibly steered the choice of applications that comply with these existing processes and roles. A nice example from the present study is ICT-supported peer support between NSCLC patients. Such an application was regarded valuable, but - from a HCPs point of view - did not fall under the responsibility of the hospitals. Third, our method of providing explicit examples of telehealthcare applications might have hampered creative processes to envision ‘out of the box’ ideas. Despite these drawbacks, our approach ensured selection of relevant treatment applications that have high chance of adoption since they fit the requirements and expectations of the target users [28].

The close cooperation with patients and HCPs gave us valuable insight into critical requirements for both the development and implementation of the telehealthcare application. Two requirements will be discussed further, because of their importance for future adoption and implementation. Patients indicated willingness in using a telehealthcare system given that results were actively used by the HCP in treatment. This is in agreement with findings from the study of Basch et al. [39]. In their study a decreased motivation in lung cancer patients for using electronic symptom-reporting was reported when the results of these reports weren’t used during consultation [39]. The influential role of HCPs on perceptions, motivation and treatment adherence of their patients regarding telehealthcare services has been previously recognized [40]. It can be expected that this influence will be present between cancer patients and their professionals as well. Especially in acute cancer care where the emotional impact of the diagnosis, together with the amount of and difficulty in understanding details of treatment and outcome, can cause high dependence on the attitudes of the HCP. This also emerged in our interviews with patients, who stated that they would do anything as long as it would help their HCP to improve treatment and recovery. Critical requirements mentioned by HCPs primarily related to their limited time available for preparation of patient consultations. Therefore, integration of the telehealthcare application with existing electronic patient records, as well as automatic merging of the ambulant data in a sensible and easy-to-view-manner was required. This requirement, that is integrating monitoring data into a coherent overview which is easy accessible by the professional, has been reported in other studies evaluating symptom monitoring [41]. Also, using a single platform that integrates all functionalities is regarded advantageous for adoption and implementation [25, 42]. Therefore, high priority was given to these requirements during design of the application. However, we also found dissimilarity in needs and requirements between HCPs. The usability study clarified that the perception if data is visualized in a ‘sensible’ and ‘easy-to-view’ manner – one of the most critical requirements – is highly individualized. For example, while pulmonologists and nurses prioritized ratings of well-being and fatigue on top of the screen, surgeons on the other hand requested to remove these outcome measures from the overview due to irrelevance for treatment. This divergence in requirements demands a flexible and modular application. So that HCPs can adapt the contents of the application based on patients’ needs or their own, personal preferences.

The co-creation in our study is thought to foster feelings of involvement and, consequently, will promote chances of acceptance and implementation. However, developing an application that works well does not guarantee successful implementation [43]. For successful implementation it is necessary to take into consideration the clinical purpose for which the telehealthcare application is used and the way the application is implemented into healthcare, also called service configuration. An example of such a service configuration, tailored to the clinical processes and context of the NKI, was described in Fig. 3 of this article. When implementing in other health care settings, the content, clinical purpose and service configuration should be validated again with HCPs and other important stakeholders. Doing so, the application will better match the clinical purpose, the service configuration and the requirements of the HCPs working at these institutes.

Several limitations of our methods should be considered. First, the results of the usability study might be biased by inclusion of ‘enthusiasts’ (that is early adopters), while unfamiliarity with technology might have prevented other patients or HCPs to participate. This may have resulted in a lower number of reported usability issues as well as a higher percentage of participants who report positive intention to use a telehealthcare application. However, we did not collect data of participants not willing to participate. Therefore we cannot draw firm conclusions to what extent bias might have influenced our results. During the following steps, reasons for non-participation should be recorded, since this can be a measure to indicate the acceptability of the application [43, 44]. Second, in our usability study participants were given minimal instructions and limited time to practice with the application. This is considered a good method to evaluate the ease of use, since it reflects the experiences of a first-time user [45]. However, it does not give insight into feasibility and adoption, such as compliance. For this, a different study method is required, during which participants will use the application for longer periods of time.

Following the staged approach suitable for development and evaluating telehealthcare [43, 46], our next step will focus on evaluating feasibility of the application with the aim to evaluate the relevance, time needed and overall feasibility for healthcare professional to use (data of) this telehealthcare application as part of post-surgery lung cancer care in larger series of patients and using controlled designs.

## Conclusions

The present study shows a positive attitude of both HCPs and resected lung cancer patients towards the use of telehealthcare applications to improve quality of current care, and to decrease insecurity of lung cancer patients about experienced symptoms, recovery, and healthy behaviour. In close cooperation with HCPs and resected lung cancer patients a new telehealthcare application was developed, consisting of a symptom monitoring module and a web based exercise module. Usability was rated acceptable, and majority of HCPs and patients were willing to adopt the application as part of regular care following lung resection. Future research is needed to optimize usability of the application, to evaluate feasibility of the application integrated as part of clinical care processes, and to optimize adoption and implementation of the application. In this process special attention is required for the crucial role of HCPs in patient compliance to telehealthcare interventions. This emphasizes the role of the HCP in the success of the application, and the need to actively involve professionals during design to promote acceptance, as well as inform and educate them during the implementation phase on how to use the system.

## Ethical approval

All procedures performed in studies involving human participants were in accordance with the ethical standards of the institutional and/or national research committee and with the 1964 Helsinki declaration and its later amendments or comparable ethical standards. According to guidelines of the CCMO, no ethical approval was needed.

## Informed consent

Informed consent was obtained from all patients included in the study.

## Consent for publication

Explicit consent for publication was obtained from the person displayed in Fig. 4.

## Availability of data and materials

Data are available on request.

## Abbreviations

EPR: electronic patient record
HCP: healthcare professional
ICT: information and communication technology
LC: lung cancer
NKI: Netherlands Cancer Institute
NSCLC: non-small cell lung cancer
SpO_2_: peripheral capillary oxygen saturation (that is, blood oxygen saturation)
SUS: system usability scale
UCD: user-centred design
UTAUT: unified theory of acceptance and use of technology
